# Parametric Imaging of Contrast-Enhanced Ultrasound (CEUS) for the Evaluation of Acute Gastrointestinal Graft-Versus-Host Disease

**DOI:** 10.3390/cells10051092

**Published:** 2021-05-03

**Authors:** Antonia-Maria Pausch, Sylvia Kammerer, Florian Weber, Wolfgang Herr, Christian Stroszczynski, Ernst Holler, Matthias Edinger, Daniel Wolff, Daniela Weber, Ernst-Michael Jung, Tobias Wertheimer

**Affiliations:** 1 Department of Radiology, University Medical Center Regensburg, 93053 Regensburg, Germany; antonia-maria.pausch@ukr.de (A.-M.P.); sylvia.kammerer@ukr.de (S.K.); christian.stros@ukr.de (C.S.); ernst-michael.jung@ukr.de (E.-M.J.); 2 Department of Pathology, University Medical Center Regensburg, 93053 Regensburg, Germany; florian.weber@ukr.de; 3 Department of Internal Medicine III, Hematology and Oncology, University Medical Center Regensburg, 93053 Regensburg, Germany; wolfgang.herr@ukr.de (W.H.); ernst.holler@ukr.de (E.H.); matthias.edinger@ukr.de (M.E.); daniel.wolff@ukr.de (D.W.); daniela.weber@ukr.de (D.W.); 4 Regensburg Center for Interventional Immunology (RCI), 93053 Regensburg, Germany

**Keywords:** GvHD, CEUS, parametric imaging, allogeneic stem cell transplantation, dynamic vascularization

## Abstract

In recent years contrast-enhanced ultrasound (CEUS) has been an emerging diagnostic modality for the detection of acute gastrointestinal (GI) graft-versus-host disease (GvHD) in patients after allogeneic stem cell transplantation. However, broad clinical usage has been partially limited by its high dependence on the expertise of an experienced examiner. Thus, the aim of this study was to facilitate detection of acute GI GvHD by implementing false color-coded parametric imaging of CEUS. As such, two inexperienced examiners with basic knowledge in abdominal and vascular ultrasound analyzed parametric images obtained from patients with clinical suspicion for acute GvHD in a blinded fashion. As diagnostic gold standard, histopathological GvHD severity score on intestinal biopsies obtained from lower GI tract endoscopy was performed. The evaluation of parametric images by the two inexperienced ultrasound examiners in patients with histological confirmation of acute GI GvHD was successful in 17 out of 19 patients (89%) as opposed to analysis of combined B-mode ultrasound, strain elastography, and CEUS by an experienced examiner, which was successful in 18 out of 19 of the patients (95%). Therefore, CEUS with parametric imaging of the intestine was technically feasible and has the potential to become a valuable diagnostic tool for rapid and widely accessible detection of acute GvHD in clinical practice.

## 1. Introduction

Allogeneic hematopoietic stem cell transplantation (allo-HSCT) represents an important and potentially curative treatment option for patients suffering from malignant or benign hematologic diseases. Fundamental to the treatment efficacy of allo-HSCT in malignant hematologic diseases is the immune mediated graft-versus-leukemia effect (GvL) mainly conveyed by donor derived T cells [1,2,3]. However, acute graft-versus-host disease (GvHD) in particular of the gastrointestinal tract poses a major life-threatening risk after allo-HSCT and substantially contributes to transplant-related mortality (TRM) [4]. Although there is an urgent clinical need for early diagnosis, it remains challenging to distinguish acute intestinal GvHD from differential diagnoses such as infectious enterocolitis, conditioning toxicity, and neutropenic enterocolitis [5,6]. Thus, treatment initiation may be delayed, which can have serious consequences for the patients due to progression of GvHD to more severe stages [5]. It has been previously shown that acute GvHD coincides with typical but rather unspecific imaging patterns, which include bowel wall thickening, abnormal mucosal enhancement, and excessive fluid-filling. In addition, extraintestinal findings such as engorgement of the vasa recta and stranding of the mesenteric fat have been reported [7,8]. Overall, imaging findings have been reported to be nearly analogous in computed tomography (CT) and magnetic resonance imaging (MRI) [9]. However, disadvantages of these approaches include nephrotoxicity of the contrast agents, exposure to a high radiation dose when performing CT, and long duration and insufficient availability in the case of MRI. In addition, since those findings are not highly specific for acute GI GvHD, it can be difficult to distinguish these patterns from common differential diagnoses [5,10]. Endoscopy with biopsy validation, which constitutes the gold standard for diagnosis of intestinal GvHD, is an invasive procedure and thus poses an additional risk for intestinal injuries [11]. In addition, histopathological analysis usually requires several days and can thus prolong treatment initiation. More recently, significant progress has been made by our group and others in establishing contrast-enhanced ultrasound (CEUS) as a diagnostic modality for acute GI GvHD [6,10,12,13]. In general, CEUS is a safe and easily accessible examination that has been extensively used for various diagnostic applications, including the evaluation of hepatic and pancreatic lesions, monitoring of renal cystic lesions, and others [14,15,16,17,18,19,20]. CEUS does not require ionizing radiation, and the microbubble-based contrast agent does not confer any risk of nephrotoxicity. Since it allows for sensitive analysis of the vascularization and perfusion of the small and large bowel, CEUS enables detection of alterations of the intestinal wall by evaluation of the dynamic microvascularization of the intestine. In this context, it has been previously demonstrated that there is barrier dysfunction of the intestine that can be illustrated by dynamic contrast-enhanced ultrasound (CEUS) during acute GvHD and that CEUS as part of a multimodal score can distinguish between mild and severe GI GvHD [6,10,12]. As such, transmural penetration of microbubbles during CEUS can be utilized as a sonomorphological correlate of tissue damage of the intestinal epithelium conferred by donor derived alloreactive immune cells [2,6]. Thus far, it has been reported, albeit with relatively low patient and control numbers, that CEUS can be a sensitive and very specific (75–100%) diagnostic tool for the detection of acute GI GvHD [5]. However, one of the major disadvantages of CEUS for the detection of acute intestinal GvHD remains that it is highly dependent on an experienced examiner and thus not widely available. To facilitate CEUS-based diagnosis of acute intestinal GvHD, we sought to implement a parametric imaging approach, which has previously been investigated in other clinical applications such as evaluation of treatment success of prostate arterial embolization or patients with prostate cancer [21,22,23]. In this proof-of-concept study, we sought to evaluate if parametric analysis of CEUS by inexperienced examiners can be utilized to detect and determine activity of acute GI GvHD, establishing it as a diagnostic tool that facilitates CEUS-based diagnosis of GI GvHD.

## 2. Materials and Methods

### 2.1. Patients

We analyzed patients who underwent allogeneic hematopoietic stem cell transplantation and were examined by abdominal ultrasound, including strain elastography and CEUS, due to clinical suspicion for acute GI GvHD as part of clinical routine. The patients then underwent endoscopy to obtain a lower GI tract biopsy, and histopathological findings were compared with ultrasound findings. The study was approved by the local ethics committee (21-225-104). Clinical grading of the patients was performed according to the standardized international scoring system [24]. The clinical course after ultrasound examination was assessed for up to 30 days. 

### 2.2. Ultrasound Examination

Abdominal ultrasound was performed as previously described [10]. In a first step, B-mode scanning in sweep technique was performed with a convex multi-frequency sector transducer (1–6 MHz, LOGIQ E9, GE, Milwaukee, WI, USA or LOGIQ S8, GE, Milwaukee, WI, USA) to reveal potentially affected areas of the small and large intestine. As such, the intestine was examined for segmental or generalized thickening of the bowel wall >4 mm, reflecting mural edema with or without concomitant intestinal free fluid. Color-coded Doppler sonography (CCDS) and power Doppler were used to screen for hypervascularized areas. Bowel loops that showed wall thickening >4 mm and edema were then scrutinized with a high resolution linear multi-frequency transducer (6–9 MHz, LOGIQ E9, GE, Milwaukee, WI, USA or LOGIQ S8, GE, Milwaukee, WI, USA). To clearly separate bowel loops with acute edema from such with chronic changes of the intestinal wall, we also performed color-coded strain elastography. All examinations were performed by an experienced examiner (more than 3000 ultrasound examinations per year for more than 20 years).

### 2.3. Strain Elastography

Color-coded strain elastography was performed in intestinal areas suspicious for acute GvHD based on B-mode findings as described before [10]. As such, elastography implemented in the high-end ultrasound machine allows assessment and visualization of elastic properties and stiffness of tissue acting against shear deformation. While examining the intestinal area of interest with B-mode ultrasound, a strain elastogram is generated by applying compression and decompression and by visualizing the resulting changes in the B-mode image. This is then transferred into a color-coded map using a scale from red (high strain, soft) to blue (low strain, hard). As a result, scar formation appears with higher stiffness coded in blue as opposed to acute intestinal inflammatory regions displayed by tissue softening coded in red or yellow [25].

### 2.4. CEUS

Contrast-enhanced ultrasound (CEUS) was performed by intravenous bolus injection of 1.5–2.4 mL sulphur hexafluoride microbubbles (SonoVue™, BRACCO, Italy) via a peripheral venous catheter followed by a 10 mL saline solution flush after written informed consent was obtained. DICOM loops up to 1 min were continuously stored, starting from the early arterial phase after 10–15 s until the late phase (up to 5 min) and were digitally stored as DICOM loops of approximately 10 s for independent retrospective reading by two inexperienced ultrasound examiners (basic experience in abdominal and vascular ultrasound) in consensus. Evaluation of the intestinal loops regarding GvHD was performed as previously described by our group. Briefly, by implementing pulse inversion harmonic imaging (PIHI) and reduced mechanical index (MI < 0.2) to exclude measurement of artifacts, penetration of microbubbles into the intestinal lumen was interpreted as manifestation of GvHD. 

### 2.5. Parametric Analysis

During the CEUS examination, DICOM loops were generated, which were then processed by the experienced examiner to generate parametric images.

This was performed with a software program integrated into the high-end ultrasound machine (LOQIC E9 or LOGIQ S8, GE, Milwaukee, WI, USA). In this regard, parametric analysis serves as a tool to visualize microbubble dynamics by color coding bubble arrival time. Using the stored dynamic DICOM loops, the point in time when the contrast media passes from mesenteric vessels to vessels of the intestinal wall was chosen. Subsequently, parametric analysis can be initiated with the built-in software, and the parametric images are generated automatically by this software. This software algorithm for interpretation of the dynamic vascularization as a built-in feature of the used ultrasound machines did not require further manual adjustments by the examiner and can thus deliver reproducible results. In this way, parametric imaging serves as an analytic tool that is based on color-coded changes of microvascularization, respectively wash-in rate. Color bars can be adjusted by the user; at our institution, we have chosen the following setup: Early arterial enhancement was coded in red, intermediate as yellow, prolonged as green nuances. Late and low enhancement was shown in blue pseudo colors. Assessment of GI GvHD in the areas of interest examined with parametric imaging of CEUS was performed based on early arterial hyperenhancement of the intestinal wall, color coded in red, and according to the interpretation of conventional CEUS images, penetration of microbubbles into the intestinal lumen shown in parametric false colors was interpreted as suspicious for acute GI GvHD. Thus, dynamic evaluation of red color signal detectable within the bowel lumen independent from moving artifacts into the lumen was applied to detect GI GvHD.

### 2.6. Ultrasound Image Analysis

The combined interpretation of B-mode ultrasound, strain elastography, and CEUS was performed by an experienced examiner (more than 3000 ultrasound examinations per year for more than 20 years). Parametric images were then generated by the experienced examiner, after the assessment of GvHD based on combined B-mode ultrasound, elastography, and CEUS had been performed. In this way, it was ensured that the experienced examiner did not include the parametric images into the GvHD assessment, and the inexperienced examiners were able to perform their evaluation solely on the parametric images and not based on the addition of the other ultrasound findings. As such, they were provided with parametric images of several different intestinal regions. These images were not preselected for the regions, where the experienced examiner found sonomorphological signs for acute GI GvHD and covered all intestinal regions that could be imaged in each patient. The evaluation of the parametric images was performed by two inexperienced ultrasound examiners with basic knowledge in abdominal and vascular ultrasound. They were completely blinded to the previously acquired ultrasound, elastography, and CEUS images. Whether microbubble penetration into the bowl lumen as a correlate of acute GI GvHD was observed or not was coded with yes/no. Observed abnormalities in bowel wall thickness on B-mode (coding: 0 (3–4 mm), 1 (5–6 mm, little), 2 (7–8 mm, moderate), 3 (>8 mm, severe)) and strain elastography were coded from 0 to 3 to assess severity (coding: 0 normal), 1 (indurated), 2 (widened with edema), 3 (homogenous soft)). 

### 2.7. Histopathological Analysis for Acute GvHD of Intestinal Biopsies

Intestinal biopsies were obtained via endoscopy of the lower gastrointestinal tract. The biopsies were processed and stained with hematoxylin-eosin. The samples were then assessed by an experienced pathologist based on the histomorphological diagnostic criteria (apoptosis, destruction of crypts, epithelial denudation) for acute GI GvHD of the Gastrointestinal Pathology Group of the German-Austrian-Swiss GvHD consortium [26,27]. The Lerner grading system was applied to determine histological severity of acute GI GvHD [28].

## 3. Results

**Figure 1:**
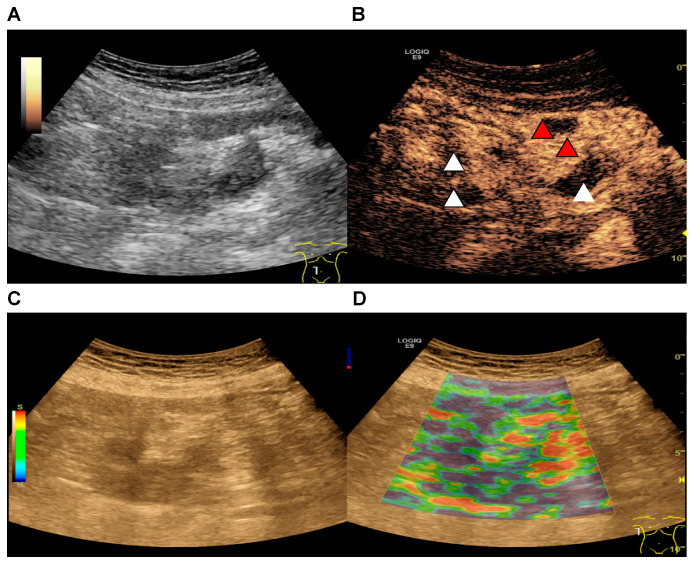
Ultrasound images of a patient with histological confirmation of acute intestinal GvHD. **A** + **C**: B-mode ultrasound showing intestinal wall thickening. **B**: CEUS image showing early arterial hyperenhancement of the intestinal wall (red arrows) and transmural penetration of microbubbles (white arrows). **D**: Soft tissue edema as a correlate of acute inflammation examined by strain elastography.

We analyzed 24 patients after allo-HSCT who underwent abdominal ultrasound including CEUS due to clinical suspicion for acute GI GvHD, with equal contribution of patients with mild and moderate to severe gastrointestinal symptoms. All patients consequently underwent lower GI tract endoscopy to obtain biopsy and had subsequent histopathological analysis. Patient characteristics are shown in Table 1. Ultrasound-based diagnosis of acute GI GvHD was either established by an experienced ultrasound examiner based on the combined evaluation of intestinal wall thickening in B-mode ultrasound, hypervascularization in color-coded doppler sonography and early arterial enhancement and transmural penetration of microbubbles in CEUS as described before [10]. DICOM loops of different intestinal regions were then used to generate parametric images, which were presented to two inexperienced ultrasound examiners with basic knowledge in vascular and abdominal ultrasound who were completely blinded to the previously acquired ultrasound and elastography images. Since acute GI GvHD often presents with segmental rather than continuous intestinal affections, the inexperienced examiners received parametric images derived from all intestinal regions that could be imaged in each patient to enable as comprehensive as possible an evaluation by parametric imaging and excluding the potential bias of presenting to the inexperienced examiners only preselected areas that an experienced examiner might image because the areas showed sonomorphological alterations suspicious for acute GI GvHD. The interpretation strategy for GI GvHD diagnosis based on CEUS with parametric imaging was focused on the occurrence of early arterial hyperenhancement color coded in red to yellow in the areas of interest and transmural penetration of microbubbles also coded in false colors according to the interpretation of conventional CEUS for GvHD. As such, Figure 1 shows representative images obtained by an experienced examiner showing intestinal wall thickening (A), transmural penetration of microbubbles (B), and soft tissue edema as correlates of acute inflammation examined by compound elastography (C and D).

**Figure 2:**
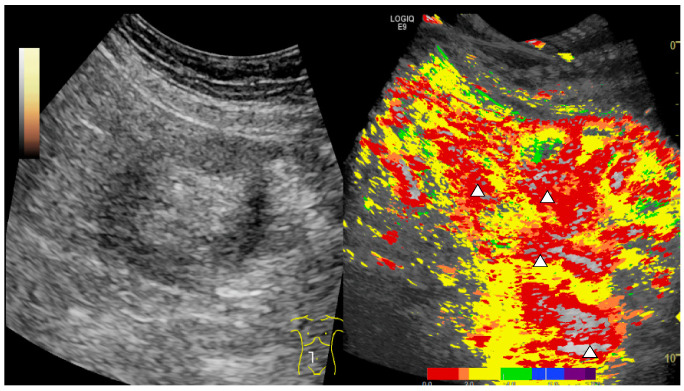
CEUS parametric imaging for the detection of acute GI GvHD. Parametric images of the same patient showing a region of the small intestine with early arterial hyperenhancement color coded in red and transmural penetration of microbubbles (white arrows).

Figure 2 depicts as a representative finding in the same patient profound early arterial hyperenhancement in an area of interest in the small intestine, which is reflected by the dominant red to yellow color coding. Importantly, parametric imaging of CEUS was also reliably able to display transmural penetration of microbubbles and color-coded representation appeared to facilitate recognition for the inexperienced examiners. 

**Figure 3:**
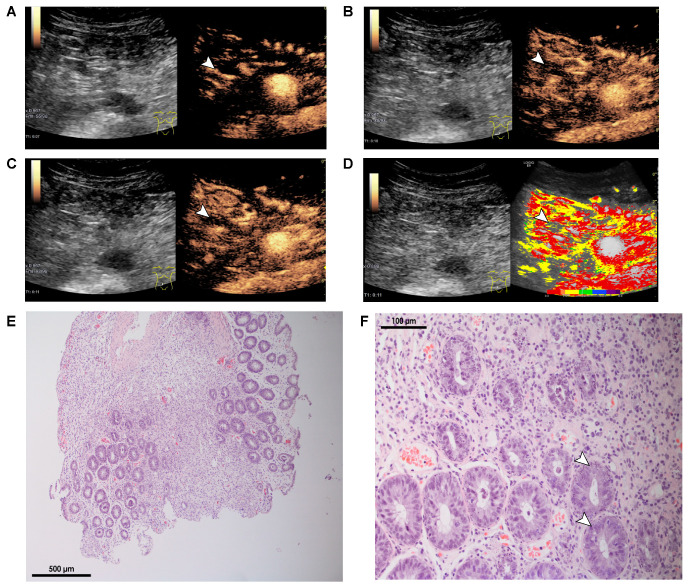
Ultrasound findings of another patient with acute GI GvHD and also an example of severe histopathological findings in GI GvHD. (**A**–**D**): B-mode and CEUS findings, including parametric imaging. Early arterial hyperenhancement of the intestinal wall (white arrows in A) and progressive transmural penetration of microbubbles (white arrows in B and C) compared with parametric images with early arterial hyperenhancement color coded in red and transmural penetration of microbubbles (white arrows). (**E,F**): Histology of colonic mucosa with severe acute GvHD (Lerner grade IV): denudation of mucosal epithelium, ulceration, and loss of crypts with abundant apoptosis in basal crypts (white arrows in F). (H&E staining, E: 50×, F: 200×).

Figure 3 gives an overview of these typical findings in CEUS, including parametric images (A–D) and an example of histopathological analysis, which shows severe acute GI GvHD (Lerner grade IV) with denudation of mucosal epithelium, ulceration, and loss of crypts with abundant apoptosis in basal crypts (Figure 3E, 50×) and vanishing crypts with numerous apoptotic bodies (Figure 3F, 200×).

Table 2 shows the diagnostic findings of the analyzed patients and the subsequent treatment for acute GvHD and the clinical outcome. Most of the patients received at least high-dose steroid therapy (2 mg/kg body weight), and a substantial fraction required additional second-line treatment. In all patients, the treatment for acute GI GvHD was initiated before the results from histology were available and thus was made based on clinical assessment and ultrasound findings.

Acute GvHD based on the Lerner classification was confirmed in 19 patients (79%) and 5 patients (21%) did not show histopathological signs of acute GvHD. In the patient group with confirmed GI GvHD, combined abdominal ultrasound examination with compound elastography and CEUS by an experienced examiner was able to correctly detect acute GI GvHD in 18 out of 19 patients (95%). In comparison, analysis of parametric CEUS images by two inexperienced examiners resulted in the correct detection of GvHD in 17 out of 19 patients (89%) (Table 3). 

**Figure 4:**
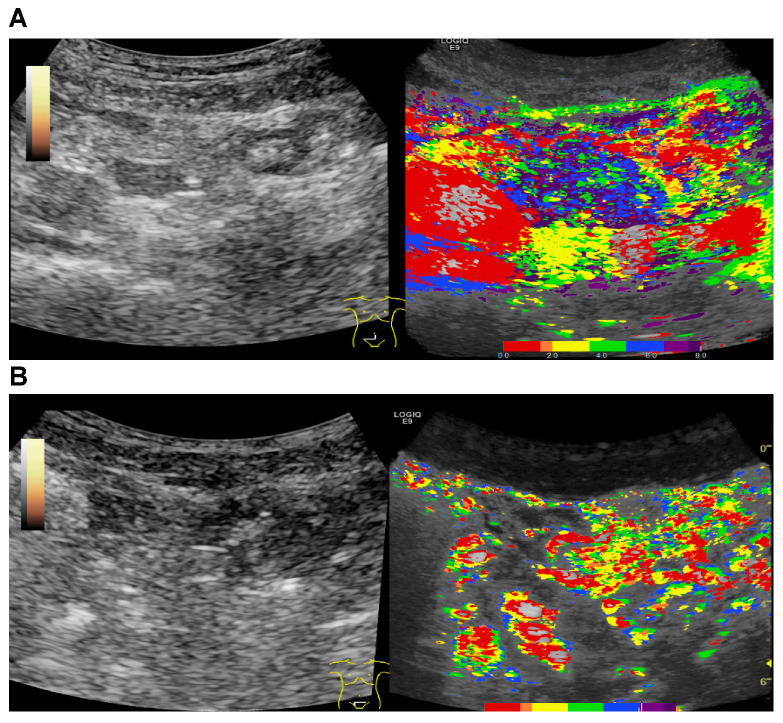
Evaluation of treatment success with parametric imaging of CEUS. B-mode and parametric images of CEUS were evaluated (**A**) before and (**B**) 4 weeks after treatment initiation. In comparison with A, B shows a regression of arterial hyperenhancement reflected by the change of color coding to the higher degree green and blue nuances and the diminished amount of red and yellow and reduced occurrence of transmural penetration.

Interestingly, as Figure 2 shows drastic early arterial hyperenhancement coded in red and clear transmural penetration of microbubbles, we also sought to test the potential of parametric imaging for evaluation of treatment success. Of note, in the four patients included in this analysis that underwent a follow-up examination after treatment initiation, we could detect regression of arterial hyperenhancement reflected by the change of color coding to the higher degree green and blue nuances and the diminished amount of red and yellow and reduced occurrence of transmural penetration (Figure 4).

## 4. Discussion

In this proof-of-concept study, we investigated for the first time the implementation of parametric false-color-coded CEUS imaging for the detection of acute intestinal GvHD, in particular by inexperienced ultrasound examiners. In this regard, CEUS has been previously reported as a diagnostic modality for acute GvHD by our group and others, and it has been demonstrated that combined sonographic methods including CEUS can distinguish mild and severe GvHD stages [6,10,12]. Since it is of high clinical importance not to delay treatment initiation of ongoing GvHD to prevent progression to more severe stages, ultrasound-based diagnosis of GI GvHD constitutes a very useful diagnostic tool. In comparison, as mentioned earlier, alternative imaging techniques such as CT or MRI potentially harbor a risk for impairing renal function due to their need for contrast agents, and their diagnostic benefit is often limited. Additionally, breathing commands and prolonged time without motion for MRI acquisition may be quite challenging for these often severely ill patients [29]. However, CEUS can be performed as a bedside examination, which is in particular favorable for compromised patients at a transplant or intensive care unit and—as opposed to CT or MRI ultrasound contrast agents—do not harbor any risk of impairing renal function and rarely show anaphylactoid reactions [25]. So far, a major limitation of CEUS has been that the interpretation is highly dependent on an experienced examiner and thus is not widely available. In this study, we addressed this limitation by implementing false-color-coded parametric imaging of CEUS. This way, using the parametric imaging display, two inexperienced examiners correctly diagnosed in 19 out of 24 patients whether acute GI-GvHD of any grade was present or not. Interestingly, this result was similar to that obtained from the conventional combined ultrasound analysis of an experienced examiner, which led to a correct diagnosis in 20 out of 24 of these patients. This study provides first evidence that parametric analysis of CEUS could be a very useful and widely accessible diagnostic tool for the detection of acute GI GvHD, enabling initiation of treatment without further delay.

Importantly, due to the pilot nature of this study, there are a few limitations to consider. These include the small number of patients and the fact that all patients initially presented with clinical symptoms for GI GvHD. Thus, the study design did not allow for sufficient analysis of false positive results, and higher numbers of patients with histological exclusion of acute GvHD would be required to assess the correct detection of true negative results. In addition, the ultrasound image acquisition and interpretation were obtained from the same operator, which might affect data reproducibility. 

Thus, further studies with a large patient cohort, including control groups without acute GI GvHD and with other forms of colitis (e.g., infectious), will be required to evaluate the exact diagnostic quality and specificity of the method, in particular compared with combined CEUS, compound elastography, and B-mode examination. Nevertheless, this study provides first evidence that parametric imaging of CEUS has the potential to substantially extend the diagnostic toolkit for the detection of acute GvHD and might be useful for easier detection of GI GvHD and clinical demonstration of CEUS findings in patients after allo-HSCT. Intriguingly, in the few patients who underwent a follow-up examination upon treatment initiation, we could clearly observe a reduction in morphological GvHD features by parametric imaging. This not only renders this method potentially useful for initial detection but also for evaluation of therapy success and raises the possibility that parametric CEUS imaging could also be useful to assess severity of GI GvHD. 

## 5. Conclusions

Despite the small number of patients included in this study, we report here for the first time that CEUS with parametric imaging in particular analyzed by inexperienced examiners has the potential to become a useful diagnostic for the early detection of acute GI GvHD in clinical routine. Thus, parametric imaging may aid in extending the use of CEUS in GvHD to eventually enable rapid treatment decisions.

## Figures and Tables

**Table 1:** Patient characteristics.

Patient Characteristcs	Value
*Patients*	24
Female, *n* (%)	7 (29)
Male, *n* (%)	17 (71)
Age, median (range)	58y (21–68)
*Diagnosis*	
Acute myeloid leukemia, *n* (%)	15 (63)
Acute lymphoblastic leukemia, *n* (%)	1 (4)
Myelodysplastic syndrome, *n* (%)	2 (8)
Myeloproliferative neoplasms, *n* (%)	1 (4)
Others, *n* (%)	5 (20)
*Conditioning regimen*	
Reduced intensity conditioning, *n* (%)	2 (8)
Standard, *n* (%)	22 (92)
*Donor type*	
Unrelated	14 (58)
Sibling	10 (42)
*Stem cell source*	
Peripheral blood stem cells, *n* (%)	20 (83)
Bone marrow, *n* (%)	4 (17)
*GvHD prophylaxis*	
Cyclophosphamide, tacrolimus, mycophenolate mofetil	10 (42)
Ciclosporine A, methotrexate, antithymocyte globuline	9 (38)
Ciclosporine A, mycophenolate mofetil, antithymocyte globuline	2 (8)
Others	3 (12)
*Acute GI GvHD grading*	
Stages I, *n* (%)	12 (50)
Stages II–IV, *n* (%)	12 (50)

**Table 2:** Diagnostic findings and clinical outcome of patients with histology.

Patient #	Days after Allo-HSCT	Overall GvHD Grade	GI GvHD Stage	GvHD Histology	B-Mode	Elastography	CEUS	PI	HD Steroids	Additional IS	Improvement in Follow-Up PI	Outcome
1	20	3	3	3	2	2	y	y	y	Eta	y	a&w
2	55	3	3	0	2	3	y	y	y	Eta	y	a&w
3	194	4	4	4	2	3	y	y	y	Eta	-	TRM (GvHD)
4	11	1	1	1	1	2	y	y	y	-	-	a&w
5	24	2	1	1	2	3	y	y	y	-	-	a&w
6	20	2	2	1	2	3	y	y	y	-	-	a&w
7	195	4	4	3	2	2	y	y	y	Eta, Eve	-	TRM
8	19	2	1	1	1	1	y	y	y	-	y	a&w
9	17	0	0	0	2	2	n	n	y	-	-	a&w
10	17	3	3	2	1	2	y	y	y	-	-	a&w
11	20	3	2	2	2	3	y	y	y	-	-	a&w
12	20	1	1	0	2	3	y	y	y	-	-	a&w
13	18	4	4	2	2	2	n	y	y	Eta, ATG	-	TRM (GvHD)
14	42	1	1	1	2	2	y	y	y	Rux	-	a&w
15	37	3	3	0	3	3	y	y	y	Eta, Rux	-	a&w
16	413	4	4	3	2	2	y	y	y	AAT		a&w
17	20	3	2	2	2	3	y	y	y	Eta	-	a&w
18	12	0	0	0	2	3	n	n	y	-	-	a&w
19	20	2	1	1	2	2	y	n	y	-	-	a&w
20	24	1	0	1	2	3	y	y	n	-	-	a&w
21	89	1	1	2	2	2	y	y	y	Eta, Rux	y	a&w
22	19	3	3	2	1	2	y	y	y	Eta	-	a&w
23	17	2	1	1	1	1	y	n	y	Eta	-	a&w
24	148	3	2	2	2	2	y	y	y	Eta, CsA	-	a&w
Median	61											

GvHD: graft versus host disease. Overall GvHD: global clinical acute GvHD grading. GI GvHD: symptom based clinical staging of GI GVHD. GvHD histology according to the histopathological Lerner grading system for acute GI GvHD (ranging from 0 (no evidence for GvHD) to 4 (histological evidence for severe GI GvHD)). B-Mode/bowel wall thickness: 0 (3–4 mm, normal), 1 (5–6 mm, little), 2 (7–8 mm, moderate), 3 (>8 mm, severe). Elastography: 0 (normal), 1 (indurated), 2 (widened with edema), 3 (homogenous soft). CEUS: contrast-enhanced ultrasound. PI: parametric imaging. y: yes; n: no. HD steroids: high-dose steroids. Additional IS: additional immunosuppression; Eta: etanercept; Eve: everolimus; Rux: ruxolitinib; ATG: antithymocyte globuline; CsA: ciclosporine A; AAT: alpha-1 antitrypsine. a&w: alive and well. TRM: transplant-related mortality.

**Table 3:** Analysis of patients with clinical suspicion for acute GI GvHD with CEUS, CEUS with parametric imaging, histopathology, and treatment decision.

GI GvHD Histology Result.		US + CEUS GI GvHD Suspicion		Parametric Imaging GI GvHD Suspicion		Treatment Initiation before Histology Result
Yes (any grade)	19	Yes	18 (95%)	Yes	17 (89%)	18
		No	1 (5%)	No	2 (11%)	(95%)
No	5	Yes	3 (60%)	Yes	3 (60%)	1
		No	2 (40%)	No	2 (40%)	(20%)

## Data Availability

Not applicable.
